# Reliability Prediction of Near-Isothermal Rolling of TiAl Alloy Based on Five Neural Network Models

**DOI:** 10.3390/ma16206709

**Published:** 2023-10-16

**Authors:** Wei Lian, Fengshan Du

**Affiliations:** National Engineering Research Center for Equipment and Technology of Cold Strip Rolling, Yanshan University, Qinhuangdao 066004, China; lianwei21@outlook.com

**Keywords:** TiAl alloy, near-isothermal rolling, neural network, exit thickness

## Abstract

The near-isothermal rolling process has the characteristics of multi-variable and strong coupling, and the industrial conditions change constantly during the actual rolling process. It is difficult to consider the influence of various factors in industrial sites using theoretical derivation, and the compensation coefficient is difficult to accurately determine. The neural network model compensates for the difficulty in determining the compensation coefficient of the theoretical model. The neural network can be trained in advance through historical data, the trained network can be applied to industrial sites for prediction, and previous training errors can be compensated for through online learning using real-time data collected on site. But it requires a large amount of effective historical data, so this research uses a combination of production data from a controllable two-roll rolling mill and finite element simulation to provide training data support for the neural network. Five trained neural networks are used for prediction, and the results are compared with industrial site data, verifying the reliability and accuracy of genetic algorithm optimized neural network prediction. We successfully solved the problem of low control accuracy of TiAl alloy outlet thickness during near-isothermal rolling process.

## 1. Introduction

Titanium-aluminiumis (TiAl) alloy a key engineering material for the future development of the aerospace and automotive industries. Its properties include low density (3.9–4.2 g/cm^3^), high elastic modulus (170 GPa at room temperature, 150 GPa at 750 °C, equivalent to GH4169), high specific strength, high specific stiffness, low coefficient of expansion, high thermal conductivity, and resistance to oxidation, creep, and fatigue [1,2]. TiAl alloy is frequently prepared using near-isothermal rolling. It is impossible to avoid difficulties with the wrinkling, cracking, and mechanical characteristics of the alloy if the rolling parameters are not well controlled and near-isothermal rolling conditions are not satisfied [3]. If we only change the material composition of the TiAl alloy without intelligently upgrading the near-isothermal rolling equipment, we cannot fundamentally solve the above problems. The finite element method has good predictive ability, but each working condition requires a lot of time and is not suitable for predicting constantly changing working conditions in industrial sites. However, neural networks can make predictions in less than 1 s, so they can play an important role in optimizing rolling plans and online monitoring.

Intelligent factories have steadily gained popularity [4,5] in recent years to overcome this issue, and realizing digital twin through simulating virtual factories has aroused great interest from all sectors of societyhas drawn significant interest from all spheres of society [6,7,8]. By using information gathered from field equipment, digital twinning can perceive the existing situation and forecast the direction of future growth throughout the rolling process. State perception is the foundation of information physics systems. The exit thickness prediction model developed in this research is a crucial component of the rolling process state perception.

Neural networks have been extensively employed in the past several decades to anticipate rolling parameters. Rolling force plays an important role in the rolling process, and the accurate prediction of rolling force is extremely important for the forming performance of sheet metal. In order to increase the accuracy of the rolling force forecast using a neural network model, Zhou et al. [9] continued the coordination adjustment to the deformation resistance and the friction force. Guo et al.’s [10] modification of the material’s deformation resistance and friction coefficient by the parameter self-adaptation approach increased the rolling force prediction accuracy. Neural networks were utilized by Li Junhong et al. [11] to forecast the mechanical characteristics of cold-rolled ribbed bars. To forecast rolling force and rolling torque under various rolling circumstances, Bisadi et al. [12] created a neural network model. Using a Bayesian neural network, Wu et al. [13] developed a mechanical model of carbon–manganese (C-Mn) steel. Fuquan Zhang et al. successfully predicted urban network traffic problems using the short-term learning ability of neural networks, which has an essential impact on the construction of smart cities [14]. Hongbo Lin et al. used a prediction model based on a convolutional neural network to successfully learn and predict the trend of stock prices [15]. In addition, many scholars at home and abroad are also studying rolling process prediction. Joon Sik Son et al. developed an online learning neural network for long-term and short-term learning, and successfully predicted rolling force data [16]. Wang, Z.H et al. established an ELM regression model for particle swarm optimization (PSO) using a dataset obtained from finite element analysis [17]. Jingyi Liu et al. proposed a rolling force prediction method based on a genetic algorithm, particle swarm optimization algorithm, and multi-hidden-layer extreme learning machine, and the prediction accuracy has been significantly improved [18]. Xie et al. improved the hybrid mathematical rolling force model and adaptive neural network to predict the rolling force of strip steel by adjusting the adaptive learning algorithm [19]. Jia et al. established the rolling force model of a tandem cold rolling mill by using the measured data and Elman dynamic recursive network method [20]. Hwang R et al. used a depth neural network (DNN) and decision tree model based on gradient lifting to accurately predict rolling force [21]. Tsang ECC et al. obtained a more reliable FNN by adjusting the knowledge representation parameter (KRP) [22]. Guo et al. modified the deformation resistance and friction coefficient of materials by using adaptive methods, improving the prediction accuracy of rolling force [10].

A large amount of research has also been conducted on the optimization of BP neural networks. Chen GY et al. [23] established a machine learning model for accurately predicting the rebound of bent pipes, which considers multiple factors such as material properties and geometric parameters. Han Lili et al. [24] increased the prediction accuracy of the width spread of the plate mill and sped up convergence by modifying the number of hidden layer neurons in the modified BP neural network. Wang et al. [25] developed a combined laminar flow control system based on a feed-forward neural network and mathematical model feedback and accurately forecasted the crimp temperature using the added momentum BP neural network model. In order to increase the model’s capacity for generalization, Li et al. [26] used BP neural networks with integrated learning to forecast the distribution of mechanical performance variations between samples.

The use of genetic algorithm (GA) for optimizing neural networks has also made good progress. A Neural Network Model Optimized by GA is a new prediction model for predicting the rolling parameters. Domestic and foreign scholars have carried out many studies to evaluate its performance in the prediction of different parameters, such as determining the metallurgical transformation of steel [27,28], predicting the rolling force and rolling moment of cold rolling [29,30], flatness prediction for hot strip mills [31], and flow stress prediction under hot deformation conditions [32,33,34]. Wouter M. Geerdes et al. combined the knowledge embedded in the heat transfer model and the temperature prediction ability of the artificial neural network: three different temperature prediction capabilities were realized, but they could only be used in an offline way and could not be monitored online [35]. Hwang, R. used machine learning methods to predict rolling force and temperature in hot rolling [21]. Azadi et al. used the finite element method to solve the governing equations of heat conduction and plastic deformation, and used a neural network model to predict the flow stress of the rolling stock [36]. Hosein Alaei et al. developed a neural network to predict the thermal expansion of work rolls during rolling, and implemented online monitoring using a validated 3D analytical model to guide and supervise the learning process [37]. Mian Jiang et al. proposed an online model for accurately predicting the thermal crown of the roll during hot rolling, and described the heat conduction process of the work roll temperature with a nonlinear partial differential equation (PDE) on cylindrical coordinates [38]. Li Cuiling et al. established a deep belief network, conducted unsupervised training on the restricted Boltzmann machine, and fine-tuned the entire network, which made the prediction accuracy better than the traditional temperature calculation formula, and the error fluctuation range was less than 8 °C [39]. YZ Zheng et al. [40] optimized the BP neural network through GA and ant colony algorithm, and deeply preprocessed the data, thereby successfully predicting the ship’s operating trajectory, RB Li [41] accurately predicted surface roughness during the turning process of nickel-based high-temperature alloys using an adaptive GA. CM Zhu [42] successfully predicted the traffic flow on the highway using a neural network model optimized by a GA, solving the problem of traffic congestion. MJ Cao [43] used a GA to predict the fatigue life of 304 stainless steel, and the predicted correlation reached over 98%. K. Tajziehchi et al. [44] used a genetic algorithm to optimize earthquake control through research on the displacement heat transfer of nanofluids with baffles.

There is no research on the prediction of exit thickness of near-isothermal rolled TiAl alloy, despite the fact that an artificial neural network (ANN) is frequently employed in the prediction of the rolling process. Compared with finite element methods, neural networks require a large amount of data for learning. Therefore, in order to train high-quality neural networks, finite element models are used to expand the training dataset, ensuring the neural network has good generalization ability [12]. The availability and accuracy of the neural networks are confirmed by contrasting the field test results with the trained neural network prediction data. After comparing the prediction outcomes of a single-layer neural network (SNN), deep neural network (DNN), deep belief network (DBN), genetic algorithm neural network (GANN), and T-S fuzzy neural network (FNN), it is confirmed that the GANN has the advantages of low calculation cost, good prediction accuracy, and robustness, and successfully solves the control precision problem of the exit thickness of near-isothermal rolled TiAl alloy. It offers a potent tool for further enhancing the mechanical qualities of TiAl alloys [3]. It also provides strong support for solving the bottleneck problem in the manufacturing of large-sized TiAl alloys.

## 2. Theoretical Model of Strip Thickness

**Figure 1:**
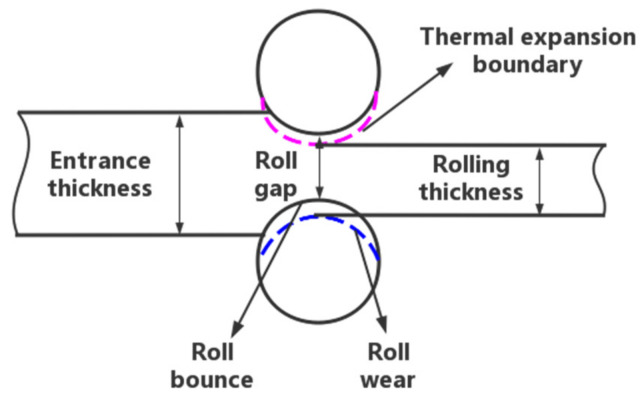
The relationship between the sub-models in the mechanism prediction model. Pink line is Thermal expansion boundary. Blue line is Roll wear.

In the hot rolling process, the rolling force of the roller on the TiAl alloy causes plastic deformation to achieve the desired target thickness. On the other hand, the TiAl alloy will also provide a reversing force to the roll, causing it to flatten elastically. This will vary the spacing between the rolls, which will modify the thickness of the finished product. Figure 1 illustrates the interaction between the compensation models in the mechanism prediction model during rolling. The following is an expression of the rolling exit thickness prediction mechanism:

(1) $$b=g-\left(\delta_{m}+\delta_{w}+\delta_{e}\right)$$

where g is the actual roll gap, and $\delta_{m}$, $\delta_{w}$, and $\delta_{e}$ are the roll bounce value, roll wear compensation, and thermal expansion compensation, respectively.

### 2.1. The Mill Bounces

The bounce equation of the rolling mill is the theoretical basis for predicting the exit thickness:

(2) $$b=g_{P}=g_{0}+\frac{\left(P-P_{0}\right)}{S}$$

where b is the exit thickness, $g_{P}$ is the gap between the loaded rollers, $g_{0}$ is the gap between the no-load rollers, S is the mill stiffness, and P and $P_{0}$ are the rolling force and zero rolling force, respectively.

**Figure 2:**
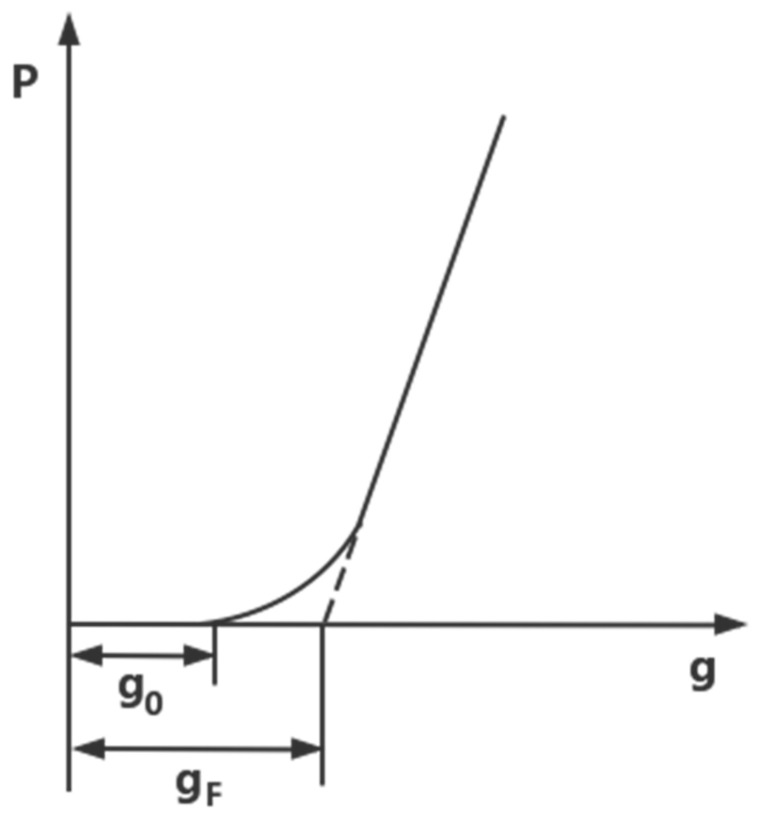
Bounce curve.

Figure 2 depicts the mill’s bounce curve. The stiffness coefficient of the rolling mill is the slope of the bounce curve’s straight portion.

The stiffness model can be expressed as follows:

(3) $$g_{P}=c_{1}+c_{2}{\left[\frac{P-P_{g}}{\Delta P}\right]}^{\frac{1}{2}}+c_{3}\left[\frac{P-P_{g}}{\Delta P}\right]+c_{4}{\left[\frac{P-P_{g}}{\Delta P}\right]}^{\frac{3}{2}}+c_{5}{\left[\frac{P-P_{g}}{\Delta P}\right]}^{2}$$

where P, $P_{g}$, and ΔP are the actual rolling force, initial rolling force value, and set the rolling force value, and $c_{1}∼c_{5}$ are the regression coefficients.

### 2.2. Mill Wear Compensation Value

Work roll wear and backup roll wear are the two main categories of mill wear. By differentiating the work roll over its length, it is possible to obtain the work roll wear model at unit speed:

(4) $$\delta_{1}=-n_{1}\left(1+n_{2}k^{n_{6}}\right)\left(1+n_{3}P_{u_{1}}^{n_{7}}\right)\left(1+n_{4}l\right)\left(1+n_{5}T\right)$$

where $n_{1}∼n_{5}$ are the correlation coefficient of roll material, $P_{u_{1}}$ is the unit rolling force, l is the contact length of the work roll, k is the relative coordinate position, and T is the temperature of the work roll.

Similarly, the wear model of the backup roll can be expressed as follows:

(5) $$\delta_{2}=-n_{6}+n_{7}P_{u_{2}}^{n_{10}}$$

where $n_{6}$ and $n_{7}$ are the correlation coefficient of roll material and $P_{u_{2}}$ is the equivalent unit rolling force.

Therefore, the mill wear compensation value at unit speed should be:

(6) $$\delta_{w}=\delta_{1}+\delta_{2}$$

### 2.3. Roll Thermal Expansion Compensation Value

The temperature of the work roll is primarily impacted by heat conduction from the TiAl alloy and cooling water during the actual production process. For the analysis, a simple model is typically employed. The following equations represent the thermal expansion of the roll in the axial *y*-coordinate direction:

(7) $$\delta_{3}=\frac{2}{r\left(1+\nu \right)\beta \int_{0}^{r}\left[T\left(R,y\right)-T_{0}\right]RdR}$$

where β is the coefficient of linear expansion, ν is the Poisson’s ratio, $T_{0}$ is the initial temperature, and $T\left(R,y\right)$ is the temperature at the coordinates $\left(R,y\right)$.

## 3. Finite Element Model

**Figure 3:**
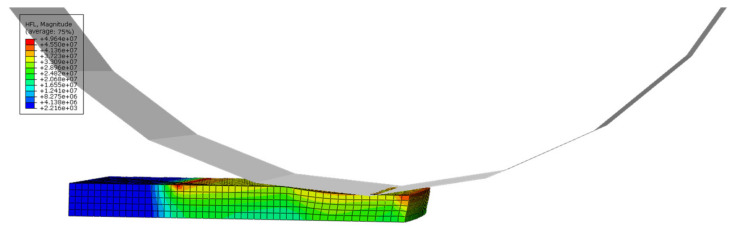
Finite element model.

The effectiveness of the finite element model established in this study was verified by comparing the exit thickness obtained from the finite element model with the experimental data. The rolled plate size is 100 × 50 × 20 mm, with a reduction rate of 20%. Due to the symmetry of rolling, a three-dimensional 1/2 model was established for finite element analysis, as shown in Figure 3.

**Figure 4:**
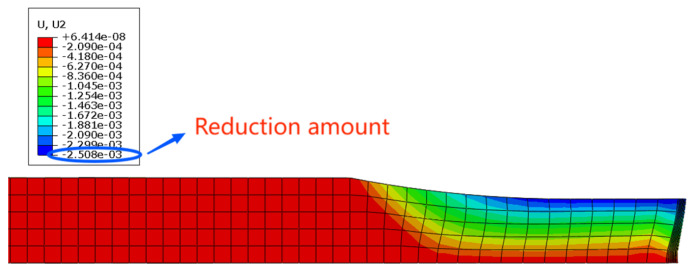
Longitudinal deformation diagram.

**Figure 5:**
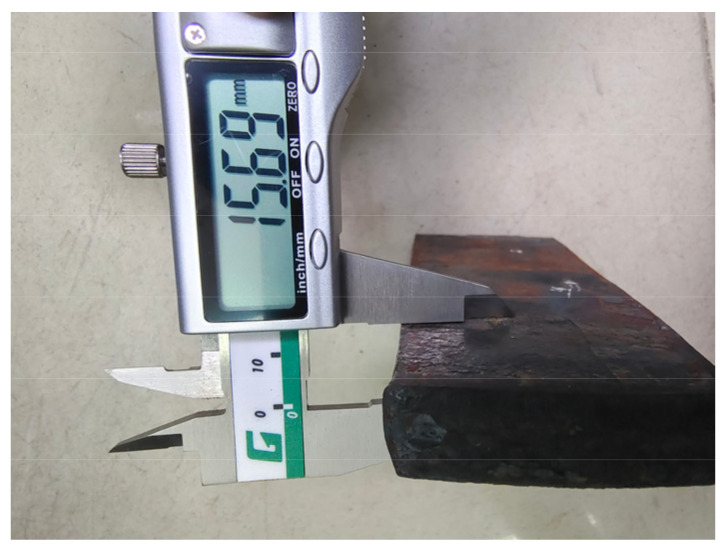
Rolling plate thickness.

From Figure 4, it can be seen that the rolling reduction is 2.5 mm. As it is a 1/2 model, the total reduction is 5 mm. The rolling exit thickness of the finite element model is 15 mm, which is only 0.69 mm different from the measured value of 15.69 mm in Figure 5, with an error of 4.4%. Therefore, the finite element model established in this study can accurately predict the exit thickness and provide data support for the neural network.

## 4. Artificial Neural Network Prediction Model

Although the BP neural network model is considered an excellent prediction model with high fault tolerance and adaptability, it also has some evident drawbacks, such as sluggish convergence speed and an easy tendency to slip into the local minimum. Different strategies are suggested to optimize the BP neural network [45] in order to address these problems.

### 4.1. Introduction to Genetic Algorithm

The study of computer simulations of biological systems gave rise to the genetic algorithm (GA). With inspiration from both Gregor Mendel’s theory of genetics and Charles Darwin’s theory of evolution, it is a stochastic global search and optimization technique designed to replicate natural evolutionary processes. It is essentially a fast, parallel, and global search strategy that can automatically gather knowledge about the search domain, adaptively controlling the search process to obtain the best answer. It is advised that readers consult the detailed principles of genetic algorithms [46].

**Figure 6:**
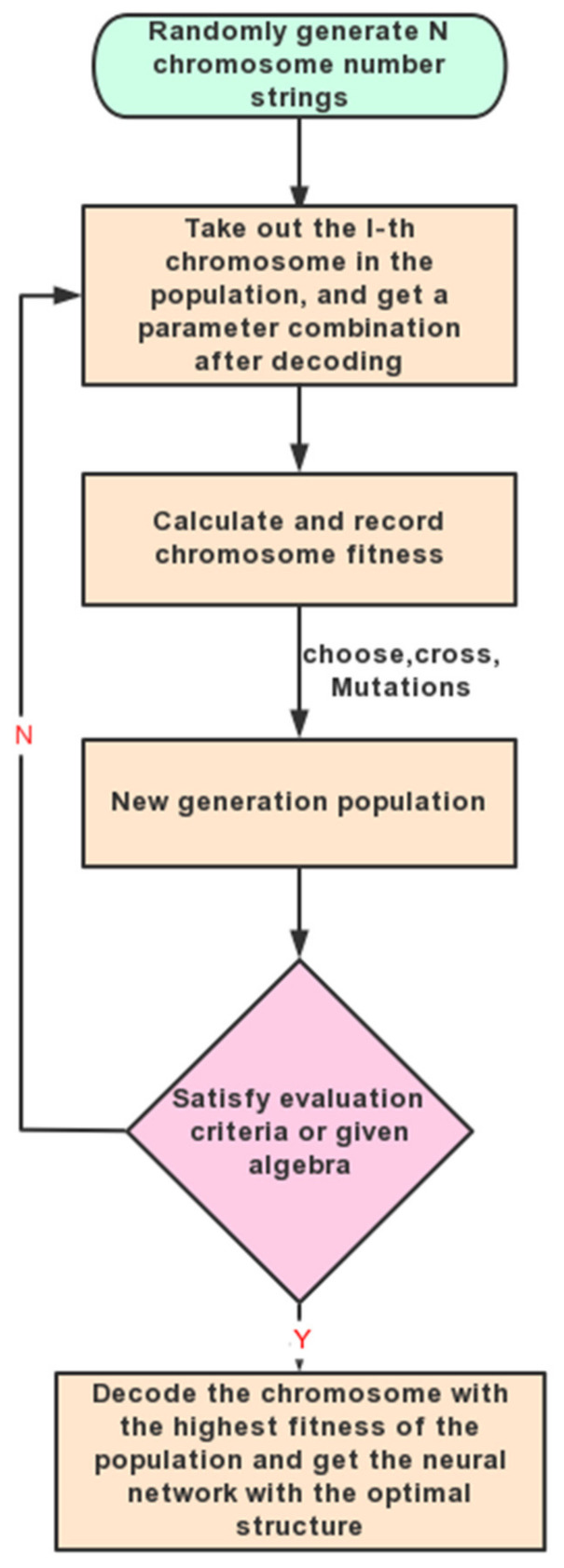
Optimization process of GA.

By using global optimization and the implicit parallelism of genetic algorithms, the optimization speed of weight coefficients can be improved. The basic process of network optimization is shown in Figure 6.

### 4.2. Determination of the Neural Network Input Parameters

A neural network’s ability to anticipate outcomes is directly determined by the relationship between its input parameters and the thickness of the rolling exit. The forecast result will differ from the real thickness if too few factors are used, and the prediction capability will be lost. The learning and training of the network will be slowed down by too many parameters, and the accuracy of the predictions will not be increased or even lowered. Therefore, choosing the right input parameters is crucial for the effectiveness of network prediction.

**Figure 7:**
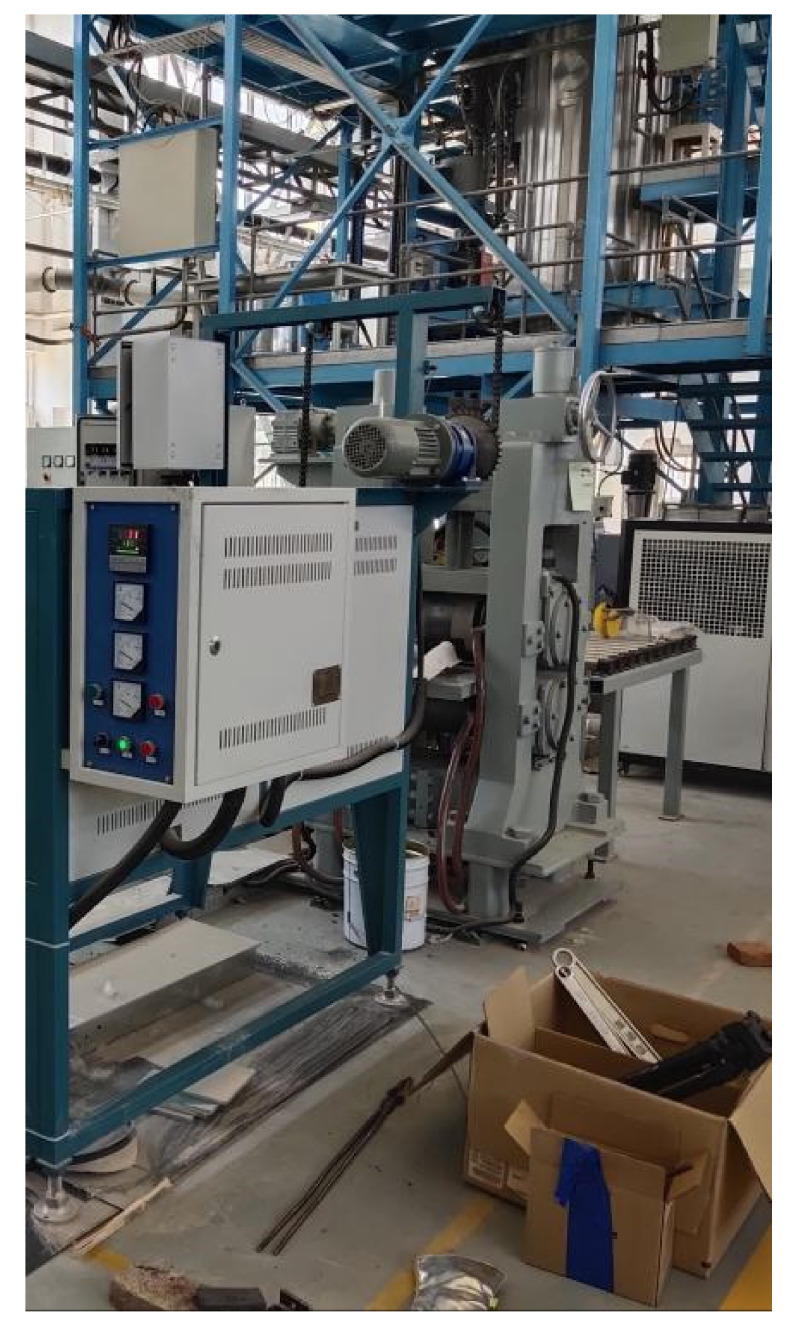
Controllable two-roll mill.

**Figure 8:**
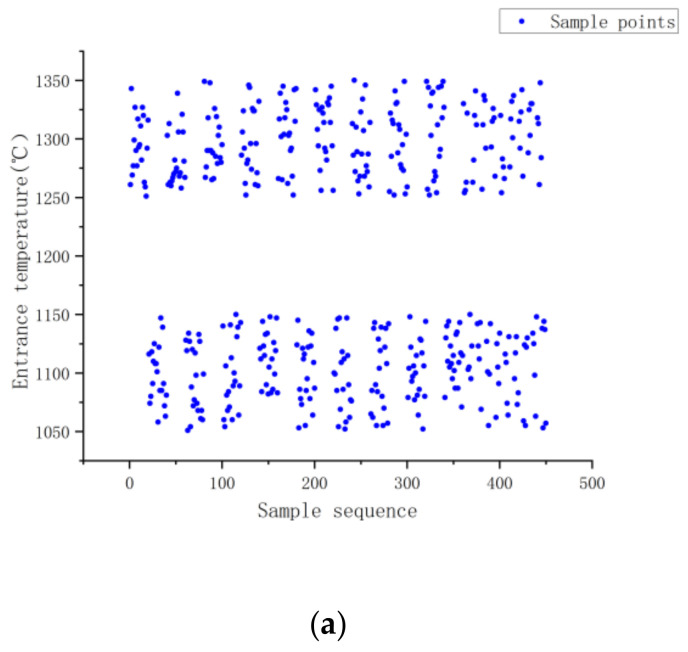

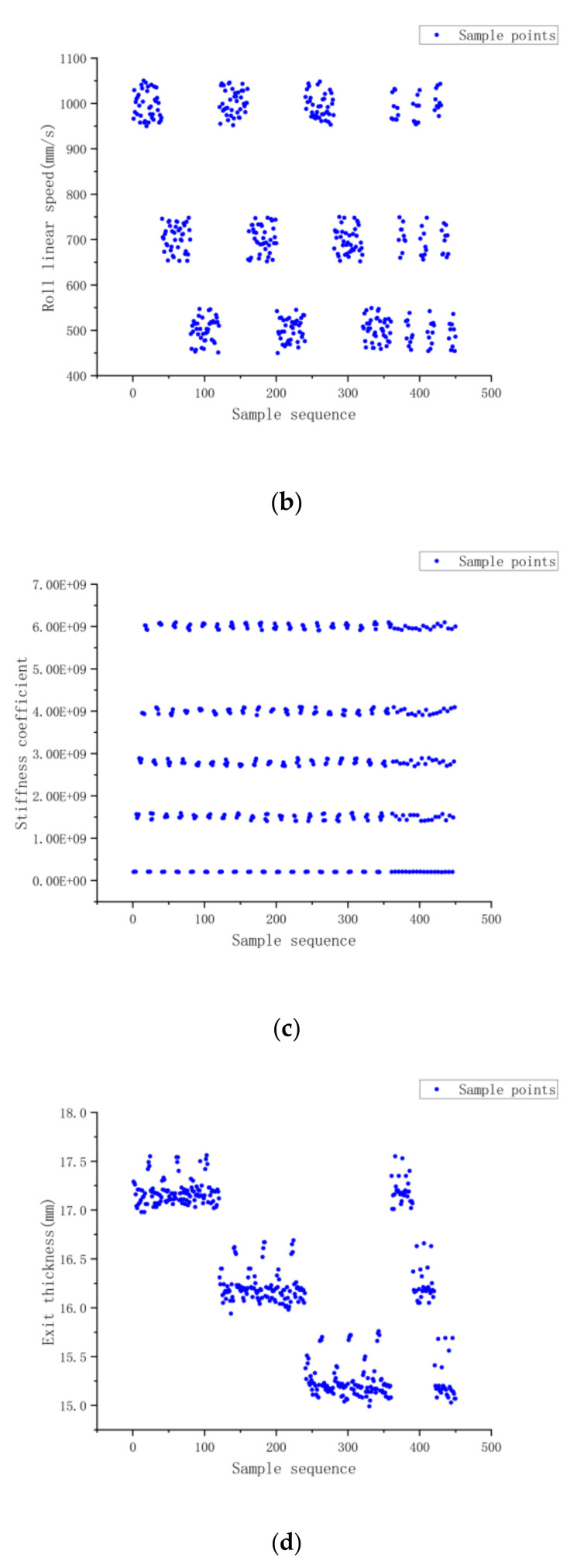
(**a**–**d**) Database scatter plot.

As indicated in Figure 7, the training data used in this research came from a controlled two-roll mill. The Abaqus program added further data. A total of 450 groups of data were gathered and the neural network was trained using 360 of those groups. Figure 8 displays the scatter plot for the database.

The correlation analysis of the reduction ratio is conducted using the GRA approach, which has been utilized extensively in recent years to choose data features for prediction models [47,48]. The correlation coefficients between each input parameter and the rolling exit thickness are listed in Table 1.

The mill stiffness and reduction ratio have a direct impact on the final thickness of TiAl alloy, while the rolling speed has an impact on the thickness through affecting the deformation resistance, oil film thickness, and friction coefficient. The inlet temperature alters the thickness of the material by modifying its resistance to deformation and altering the rolling force necessary for plastic deformation.

### 4.3. Determination of the Number of Hidden Layer Units in Neural Network

The input layer, hidden layer, and output layer make up the neural network. The number of hidden layer units directly affects the ability of neural networks to predict the thickness of the rolled TiAl alloys, as, once the input parameters are established, the number of units in the input and output layers can be determined. A process of trial and error is used in this study to assess the hidden layer unit count from 1 to 10. Table 2 displays the overall error E.

Table 2 shows that, when the number of hidden layers grows, the overall prediction error initially drops and subsequently increases. When there are six elements in the hidden layer, the overall error is at the lowest level. The transfer functions of the five neural networks are shown in Table 3.

## 5. Analysis and Discussion of Prediction Model

**Figure 9:**
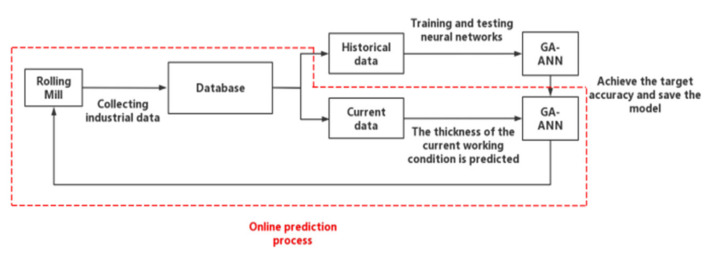
Predictive flow chart.

**Figure 10:**
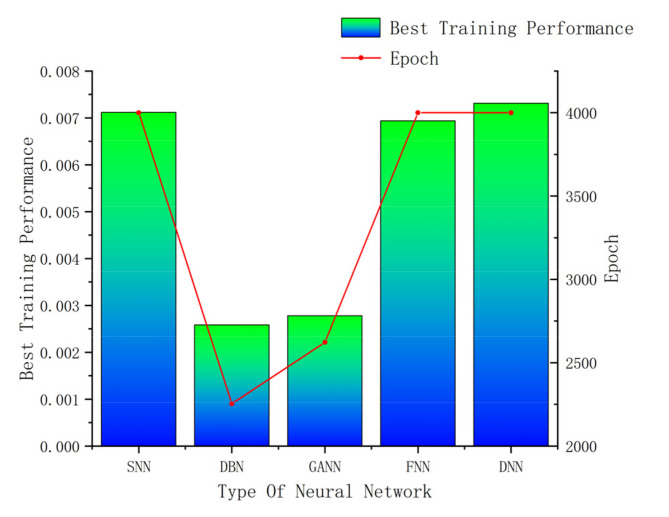
Training performance comparison.

The root mean square error (RMSE) results predicted by the five algorithms are shown in Table 4. The execution time is shown in Table 5. The prediction process of outlet thickness is shown in Figure 9. If the training error reaches the set value or the training frequency reaches the upper limit, the training will be stopped, as shown in Figure 10.

**Figure 11:**
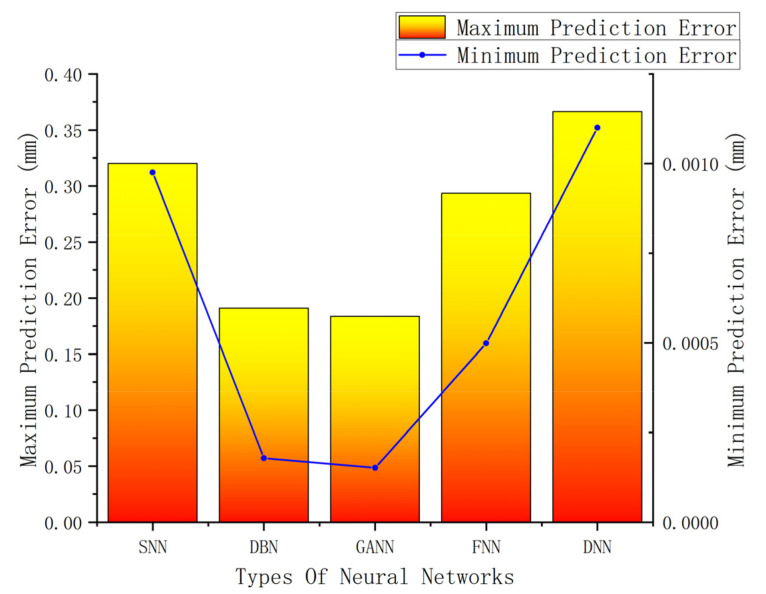
Maximum and minimum prediction error distribution.

**Figure 12:**
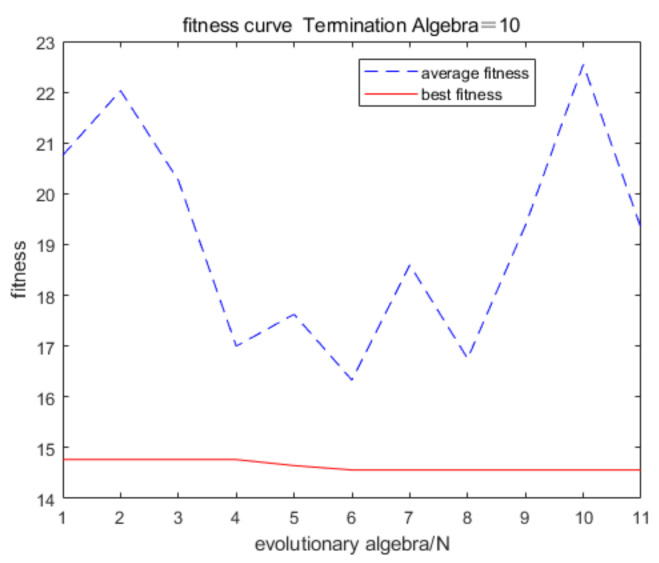
Fitness curve.

**Figure 13:**
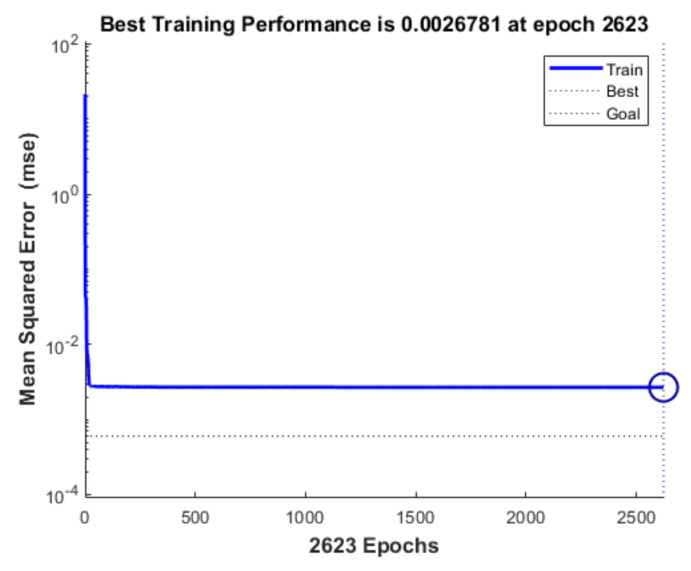
GANN model training process.

**Figure 14:**
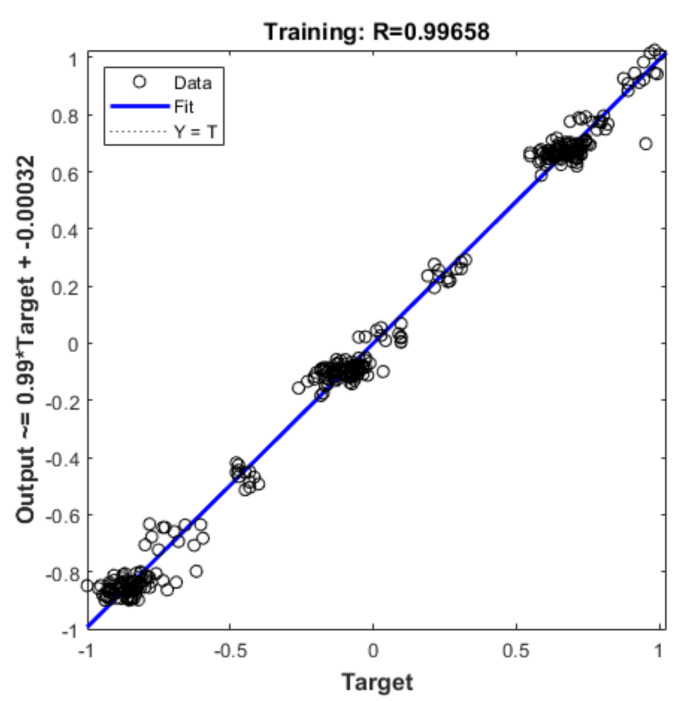
GANN model regression curve.

**Figure 15:**
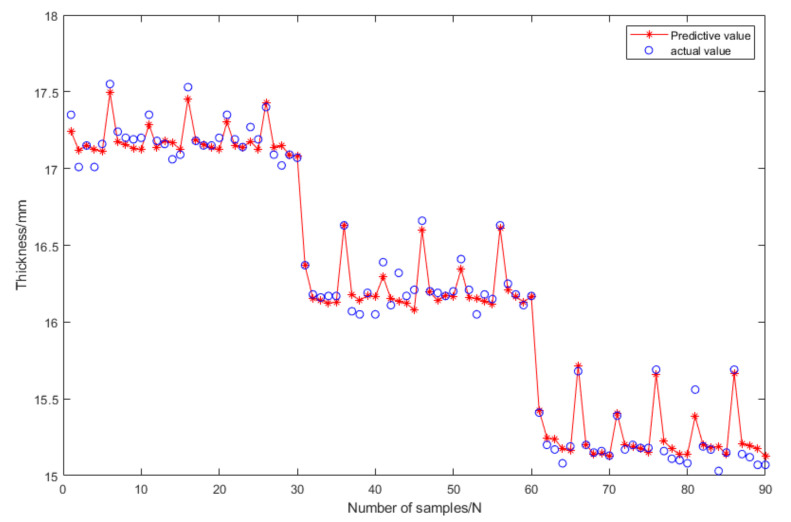
GANN model prediction curve.

Figure 11 displays a comparison of the five models’ errors. From Figure 10 and Figure 11, it can be seen that, although the training performance of the DBN is slightly better than that of the GANN, the GANN performs better in terms of prediction accuracy. Table 6 provides an overview of the greatest forecast error and overall forecast error. It is clear that the FNN model has the biggest prediction error. Although the errors of the DBN and GANN models are comparable, the GANN model is better able to avoid the local minimum issue with the DBN model. The GANN model’s maximum forecast error is merely 0.18 mm, and its average prediction error is 0.05 mm. Figure 12 depicts the fitness curve for the GANN model, which has a fitness curve termination algebra of 10 generations. Figure 13 depicts the mean square error diagram for the GANN model during training, with the ideal performance value being 0.0026781 and approaching it quickly. Figure 14 displays the distribution of the predicted values along the target line, with a correlation coefficient of 0.99658. In Figure 15, the prediction curve is displayed. The GANN model uses a 4-6-1 network configuration. The transfer functions of the hidden layer and the output layer are tansig and purelin, respectively. The population size is 10 generations, the cross probability is 0.2, and the mutation probability is 0.1 in the evolutionary algebra.

**Figure 16:**
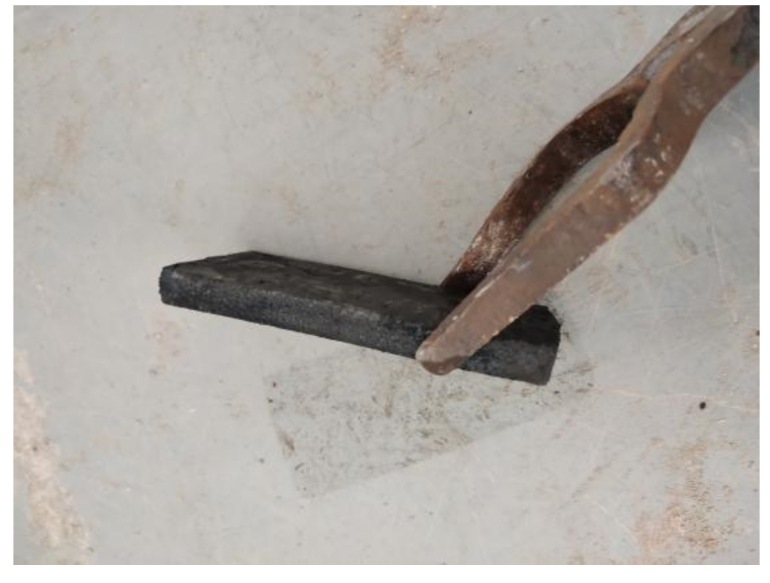
TiAl alloy specimen after near-isothermal rolling.

The near-isothermal rolling exit thickness of the TiAl alloy is accurately predicted using the GANN model, and the rolling parameters are tuned to prevent cracking. The rolling effect is depicted in Figure 16. In conclusion, the TiAl alloy exit thickness during rolling may be predicted using the GANN model.

## 6. Conclusions

This article predicts the outlet thickness of near-isothermal rolled TiAl alloy using five neural networks, and after comparison, the following conclusions are drawn:

(1) After comparison, the GANN model has high prediction accuracy, with a maximum prediction error of only 0.18 mm, an average prediction error of 0.05 mm, and a root mean square error of 0.0064;
(2) The GANN model has the function of online monitoring, with a training time of 1.8457 ± 1.2359 s and a testing time of 0.01284 ± 0.01157 s;
(3) As the rolling speed increases, the entrance temperature increases, the stiffness coefficient increases, and the outlet thickness decreases;
(4) Neural networks have the characteristic of fast response, and using this powerful tool to create more intelligent devices for human society will have a significant impact on the future.

## Figures and Tables

**Table 1:** Correlation degree of the input parameters.

Input Parameters	Gray Relation
Rolling speed	0.984856
Inlet temperature	0.986253
Stiffness Coefficient	0.995423
Reduction rate	0.99867

**Table 2:** The relationship between the total prediction error and the number of hidden layer units.

Number of Hidden Layer Elements	Total Error
1	1.1974
2	0.7901
3	0.5174
4	0.5815
5	0.4809
6	0.4458
7	0.5813
8	0.5776
9	0.5702
10	0.5977

**Table 3:** The transfer functions.

Algorithm	Transfer Functions
SNN	Compet-logsig-poslin
DNN	Purelin-radbas-logsig
DBN	Satlins-purelin-logsig
GANN	Trainlm-tansig-purelin
FNN	Radbas-satlins-compet

**Table 4:** RMSE results of five algorithms.

Algorithm	RMSE
SNN	0.0168
DNN	0.0154
DBN	0.0132
GANN	0.0064
FNN	0.0153

**Table 5:** Execution time of 5 neural networks.

Algorithm	Training Time (s)	Testing Time (s)
SNN	1.8569 ± 1.3547	0.01352 ± 0.01536
DNN	7.6524 ± 3.6528	0.02549 ± 0.01626
DBN	50.3521 ± 5.2169	0.01529 ± 0.01752
GANN	1.8457 ± 1.2359	0.01284 ± 0.01157
FNN	10.5821 ± 2.5674	0.02463 ± 0.01784

**Table 6:** Prediction error.

Algorithm	Max Prediction Error (mm)	Total Forecast Error (mm)
DBN	0.1911	4.5776
DNN	0.2934	8.0541
SNN	0.3203	8.0829
GANN	0.1838	4.4577
FNN	0.3191	8.9736

## Data Availability

Some data have been provided in the research, while other data can be obtained by contacting the first author.
